# Root Cause Analysis of Omissions and Delays in the Initiation of Neoadjuvant Chemotherapy in Eligible Patients with Breast Cancer in British Columbia, Canada

**DOI:** 10.3390/curroncol33020087

**Published:** 2026-02-01

**Authors:** Jonathan L. H. Chan, Jaimie J. Lee, Hyejee Ohm, Kathryn V. Isaac, Alan Nichol

**Affiliations:** 1 Department of Surgery, University of British Columbia, Vancouver, BC V5Z 1M9, Canada; jchan918@student.ubc.ca (J.L.H.C.); jaimie.lee@ubc.ca (J.J.L.); kathryn.isaac@ubc.ca (K.V.I.); 2 Department of Radiation Oncology, BC Cancer, Vancouver, BC V5Z 4E6, Canada; 3 Division of Medical Oncology, University of British Columbia, Vancouver, BC V6T 1Z3, Canada; hyejee.ohm@bccancer.bc.ca

**Keywords:** neoadjuvant chemotherapy (NACT), breast cancer, root cause analysis, treatment omission, treatment delay, medical oncology

## Abstract

Patients with high-risk breast cancers may benefit from receiving neoadjuvant chemotherapy (NACT) to reduce tumour size and allow for breast conserving surgery. Response to NACT also informs prognosis and broadens adjuvant treatment options. According to international guidelines, NACT should commence within 28 days of diagnosis. We conducted a retrospective chart review of patients eligible for NACT at a Canadian provincial cancer centre to determine the incidence and root causes of omission or delay in initiation of NACT. Of 100 patients eligible for NACT, 73 received it, while 7 were not referred to medical oncology for consideration of NACT. Of the 73 patients who received NACT, only 15 (21%) started treatment within 28 days of diagnosis. The median diagnosis-to-NACT wait time was 40 days [IQR 30–53]. Of 54 delayed cases, 39 (72%) were due to patients waiting 21 days or more for a medical oncology consultation. Lack of, or delays in medical oncology consultation are the most prominent causes of both NACT omissions and delays. Improving triage of patients potentially eligible for NACT to medical oncology may be an effective intervention to improve patient outcomes.

## Simple Summary

Neoadjuvant chemotherapy (NACT) can reduce the extent of surgery and improve outcomes for patients with high-risk breast cancers. Chemotherapy should be started shortly after diagnosis to be most effective. However, steps between diagnosis and starting NACT can prevent patients from receiving treatment and impose delays. In this study, we aimed to find reasons leading to omission or delays in receiving NACT in eligible patients. Seventy-three percent (73/100) of eligible patients received neoadjuvant chemotherapy. However, of these patients, only 21% (15/73) started chemotherapy within 28 days of diagnosis, with patients waiting a median of 40 days [IQR 30–53] from diagnosis to starting NACT. Referral to medical oncology was identified as the key obstacle to neoadjuvant chemotherapy receipt. Improving triage to medical oncology for patients eligible for NACT could improve their outcomes.

## 1. Introduction

Neoadjuvant chemotherapy (NACT) may be indicated for patients with high-risk breast cancers. Consideration of NACT is particularly important for those with triple-negative and human epidermal growth factor 2 (HER2)-positive breast cancers, which are aggressive and pose high risks of relapse and death [1,2]. NACT may help downstage large tumours, allowing for breast conservation and sentinel lymph node biopsy [3,4,5]. Smaller and shorter surgeries decrease the risk of post-operative complications [6,7,8].

Additionally, response to NACT informs adjuvant treatments and patient prognosis. Lower residual cancer burden (RCB) scores after NACT are correlated with improved relapse-free survival rates [9,10]. Patients who achieve pathological complete response (pCR), defined as complete clearance of cancer following NACT, have excellent prognoses. In a multicentre analysis by Yau et al., patients with hormone receptor-positive, HER2-positive, and triple-negative breast cancer achieving pCR had 91% event-free and 93% distant relapse-free survival rates at 5 years, compared to 58% and 60% for patients with RCB-3 [9]. Regional radiotherapy may also be avoided among patients achieving pCR without impacting survival [11]. For patients with residual disease after NACT, adjuvant treatment can be escalated to improve survival, as per the CREATE-X and KATHERINE trials [12,13]. 

Clinical guidelines recommend that patients should commence NACT within 28 days of diagnosis [14]. Delays to NACT may worsen patient outcomes. De Melo Gagliato et al. found a 1.28-fold increase in death risk in patients who received NACT more than 61 days after diagnosis, compared to those who received it within 30 days [15]. Similarly, Hatzipanagiotou et al. suggested that delays in NACT beyond 42 days after diagnosis may reduce overall survival [16].

Unfortunately, various bottlenecks in the current care pathway in British Columbia (BC) can introduce barriers to accessing NACT, potentially leading to omission or delay of treatment. Despite previous research indicating shorter time to treatment with multidisciplinary care teams [17], oncologic care in BC remains fragmented. Referral processes for different oncologic specialties are separate, necessitating multiple referral processes before patients can be seen for consideration of NACT. Improvements can only be achieved if specific chokepoints in the system inhibiting access to NACT can be identified and adequately addressed.

The most recent data available in BC, between 2013 and 2018, indicated that 82% of patients started NACT within 31 days of diagnosis [18]. More recent data on NACT timing, as well as data on omission of NACT among eligible patients with breast cancer, were unavailable. Previous research has identified major obstacles in breast cancer care at a regional hospital in BC [19], but, to our knowledge, no provincial or national analysis of NACT access specifically has been done.

The purpose of this study was to identify the prevalence of timely NACT receipt among eligible patients with breast cancer in BC, as well as to determine key reasons for omission and delay of NACT among eligible patients.

## 2. Materials and Methods

We performed a retrospective chart review of patients who received a core biopsy for breast cancer at a hospital in BC between 1 January 2024, and 31 December 2024, and whose data were available in our electronic medical records (EMRs) system. A list of all breast core biopsies performed within BC in 2024 was obtained from the BC Cancer Registry, forming a list of potentially eligible patients. From this list, patients were included in the study if they met inclusion and exclusion criteria until the enrollment goal was reached. The inclusion criteria were diagnosis with either triple-negative or HER2-positive invasive ductal or lobular carcinoma, 80 years old or younger at the time of diagnosis and presenting with stage II or III disease based on clinical examination and imaging according to the American Joint Committee on Cancer Breast Staging Manual, 8th edition [20]. Patients with stage I or IV cancer, as well as subtypes other than ductal or lobular cancer, were included if they started NACT with curative intent. Patients were excluded if they had any previous personal history of breast cancer, presented with bilateral disease, or if their clinical staging information or cancer subtype could not be ascertained from the EMR. Ethics approval was obtained from the Research Ethics Board of the University of British Columbia/BC Cancer (application number H24-02719). 

We collected demographic information, including age at diagnosis, city of residence, and BC Health Authority for the receipt of medical care. Tumour information was recorded, including estrogen, progesterone, and HER2 receptor status, histologic grade, size on imaging, presence of suspicious lymph nodes by palpation or on imaging, and clinical stage. Treatment status was recorded, including whether the patient received breast surgery, NACT, adjuvant hormone therapy, or supplemental adjuvant chemotherapy, and whether the provider recommended NACT (if applicable). Visit information was collected, including the dates of first consultation with both surgical and medical oncology, as well as date of first imaging, diagnosis, surgery, and commencement of NACT. For patients receiving NACT, post-treatment T and N stages were collected to ascertain their stage post-NACT, and whether pCR was achieved. Additionally, to determine potential causes of delay, the date of genetic testing was collected if applicable.

From the collected data fields, the times between diagnosis and first consult, diagnosis to first treatment, and first consult to first treatment were calculated for each patient. Diagnosis was defined as the date of collection of the first biopsy showing malignancy. If applicable, times were calculated for both types of consultations (surgical or medical oncology) and both types of treatment (surgery and NACT). The median and interquartile range were calculated based on data from all patients.

The charts of all patients theoretically eligible for, but who did not receive, NACT, as well as all patients starting NACT more than 28 days after diagnosis, were reviewed to identify the main reason for omission or delay of NACT. Reasons for omission were broadly grouped into four categories, including patients declining chemotherapy, NACT not being offered, patient not being offered an appointment with medical oncology, and other reasons not falling into one of the three aforementioned categories. Reasons for delay were similarly grouped into four categories, including delayed medical oncology consultation (more than 21 days after diagnosis), patient preference to delay treatment (defined by any provider note explicitly mentioning that the patient would like to delay starting NACT for any reason), unspecified patient factors (any new illness delaying the start of NACT from original treatment plan), and unspecified provider causes (any reasons mentioned in provider notes as delaying the start of NACT, for example, additional imaging needed). All patients waiting more than 21 days between initial diagnosis and date of medical oncology consultation were classed as experiencing delays in medical oncology consultation. This is because steps required between consulting with medical oncology and beginning NACT, such as tests and scheduling, typically require at least seven days in BC.

Correlations between variables potentially contributing to delayed NACT initiation and time to chemotherapy initiation were determined using either Spearman correlation coefficients, for continuous variables, or Mann–Whitney U tests, for discrete variables. Significance was defined to be *p* = 0.05. All tests were two-tailed. Spearman rank coefficients were calculated using Microsoft Excel version 2512, while Mann–Whitney U tests were performed using R version 4.4.2 on RStudio version 2024.12.1+563.

## 3. Results

### 3.1. Study Population

**Figure 1:**
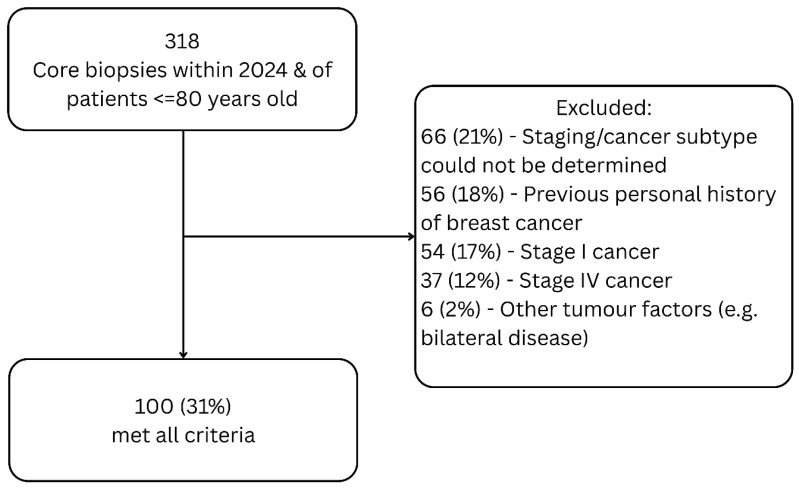
Flowchart of study inclusion and exclusion.

After initial screening, 318 patients met inclusion criteria. Following full chart review, 66 patients (21%) were excluded as staging or cancer subtype could not be determined from available charts. Fifty-six patients (18%) were excluded due to previous history of breast cancer. Further, 54 (17%) and 37 patients (12%) were excluded due to presenting with stage I and IV disease. Six (2%) patients were excluded due to other miscellaneous reasons, including presenting with bilateral disease or cancer subtypes other than ductal or lobular carcinoma. In total, 100 patients (31%) were included in the study. Figure 1 provides a flowchart of study inclusion and exclusion.

All 100 patients were eligible for NACT based on their tumour characteristics, or were borderline cases explicitly judged by their care team to likely benefit from NACT. One stage I patient received NACT as tumour size was exactly 20 mm on imaging, 1 mm short of classification as a stage II tumour; medical oncology determined that this patient was likely to derive benefit from receiving NACT. A stage IV patient with distant metastasis only to mediastinal lymph nodes was also included as the patient was treated to all sites of disease with curative-intent radiotherapy. Baseline demographics of the study cohort are shown in Table 1.

### 3.2. Treatment Status

The NACT treatment status of all patients in the study is detailed in Table 2. After meeting with medical oncology, 76 patients were offered NACT, with 73 proceeding to treatment. All patients reviewed were offered surgery, though one patient declined all interventions.

### 3.3. Reasons for Omission of NACT (n = 27)

A total of 27 patients eligible for NACT based on staging and biomarker profile did not receive it, with reasons for omission outlined in Table 3. Two patients declined NACT. NACT was not offered to nine patients after weighing the benefits versus risks, while another patient was not eligible for NACT due to having received “alternative therapy” containing chemotherapy agents outside standard of care. Of the 17 patients not offered NACT, 12 had HER2-positive disease and 5 had triple-negative breast cancer. Seven patients were not offered a consultation with medical oncology before surgery, with two cases each in the Coastal, Fraser, and Island health regions and one in the Northern health region. Additionally, one patient explicitly declined all systemic therapy upfront and therefore did not meet with medical oncology. One patient sought treatment out-of-country due to patient–provider misunderstandings and therefore did not receive NACT.

There was a significant difference in age between those who received NACT and those who did not (*p* = 0.0003). The median age of patients in the study cohort who received NACT was 54 [IQR 41–63], compared to a median age of 64 [IQR 58.5–73.5] among those who did not receive NACT.

### 3.4. Treatment Timeframes

The median wait from diagnosis to first consultation, either medical oncology or surgical, was 16 days [IQR 11–26], then an additional 20 days [IQR 14–30.5] to first treatment. The median wait time from diagnosis to first medical oncology consultation was 28 days [IQR 20–49]. The median time from diagnosis to commencing treatment was 40 days [IQR 30.3–53.8]. Median timeframes between different key steps in the treatment pathway are shown below in Table 4.

### 3.5. Reasons for Delay in NACT (n = 54)

**Figure 2:**
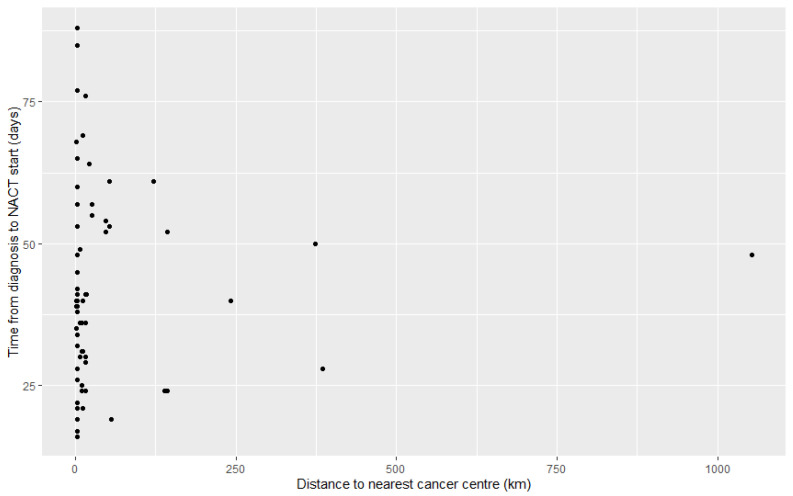
Comparison between distance to nearest cancer centre from patient city of residence and diagnosis-to-chemotherapy time.

Out of 73 patients who received NACT, 54 (74%) initiated it more than 28 days after initial cancer diagnosis. Of these, 72% (39/54) had an initial consult with medical oncology 21 days or more after diagnosis, which was defined as a delayed medical oncology appointment. One patient delayed treatment due to travel. One patient delayed NACT due to a concurrent illness, while another patient experienced a delay due to further imaging results being needed. Reasons for delay were unclear on 12 of 54 patient charts (22%), with no data in the chart supporting other reasons such as language barriers or other socioeconomic factors. Breakdown of reasons for delayed NACT are shown in Table 5. As shown in Figure 2, no correlation was found between patient distance to nearest cancer centre and time between diagnosis and commencing NACT, with a Spearman correlation coefficient of 0.1. 

Of the patients who received NACT, 64% (47/73) underwent genetic testing before surgery, compared to 36% (26/73) who did not (Table 6). A Wilcoxon test revealed no correlation between genetic testing status and time from diagnosis to starting NACT (*p* = 0.9).

**Figure 3:**
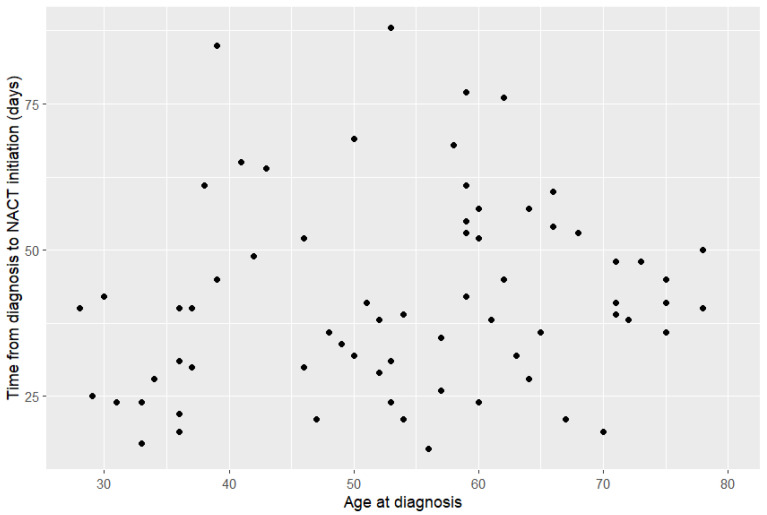
Comparison between age at diagnosis and diagnosis-to-chemotherapy time.

Similarly, no correlation was found between age and time to initiating NACT, with a Spearman correlation coefficient of 0.2 (Figure 3).

## 4. Discussion

This study analyzes the treatment status and timing of a recent cohort of patients with high-risk breast cancers in BC, identifying reasons leading to omissions and delays in NACT. In our review, 73% of patients (73/100) eligible for NACT based on tumour and biomarker profile received it. Of the 27 patients who were otherwise eligible but did not receive NACT, at least 7 (26%) were not offered a medical oncology consult before surgery, and therefore were not considered for NACT. Clearer provider–patient communication may also reduce incidence of NACT omission, as one patient in this review did not receive NACT due to a misunderstanding that treatment would not be offered in Canada, thus seeking treatment out of country. No other identifiable causes of NACT omission were actionable—for example, cases where patients were unlikely to benefit from NACT or where patients declined treatment.

Previous research has identified potential explanations for the lack of referral to medical oncology. Yee et al. (2024) reported regional variations in referral practices as a potential confounder in whether patients see medical oncology for consideration of NACT [21]. In contrast, we observed no difference in lack of referral to medical oncology between different health regions in our study. Additionally, delays in biomarker testing to determine high-risk status may prevent or delay referral to medical oncology, and therefore NACT access for eligible patients [19].

A large majority of patients (93%) were seen by medical oncology to consider NACT, whether within or outside the clinically recommended timeframe. Previous research from Ontario found that only around one-in-three patients with stage II or III HER2 or triple-negative breast cancers received chemotherapy first [22], compared to 93% in this study. However, the lack of referral to medical oncology for the remaining 7% (7/100) of eligible patients is still concerning and should be further reduced.

Age was identified as having a statistically significant correlation with omission of NACT (*p* = 0.0003). The median age of those who did not receive NACT was 64 [IQR 58.5–73.5], 10 years older than those who did receive NACT. However, this includes patients who omitted NACT due to medical oncology determining that the risks of chemotherapy outweighed its benefits. The median age for this group of patients was 67 years old [IQR 56–77], higher than the median study cohort age of 58.5 [IQR 45.3–66]. Older patients are more likely to have comorbidities that increase the risk of receiving NACT [23], limiting any interpretation of causation from this correlation.

Notably, only 21% of patients (15/73) receiving NACT started treatment within 28 days of diagnosis, as recommended by clinical guidelines. The median time from diagnosis to starting NACT was 40 days [IQR 30–53], 12 days more than the maximum recommended in clinical guidelines. Further, 71% (39/54) of delays in commencing NACT could be attributed to delays of 21 days or more to a medical oncology appointment. As the median time between first medical oncology consult and commencing NACT was 11 days [IQR 7–16], patients who do not meet with medical oncology before the 21st day post-diagnosis were unlikely to begin treatment within the next 7 days.

Various resource constraints can prolong the timeline from diagnosis to commencing NACT, for example, delayed biopsy, barriers to accessing staging imaging, and shortage of chemotherapy chairs or medical oncologists to consult with patients. While the wait time to receive NACT is conventionally measured from the time of biopsy, long wait times to biopsy still delay receipt of NACT and should be considered. Furthermore, in BC, patients are usually sent for staging imaging at the same time as chemotherapy workup, with distant metastasis presumed absent (i.e., eligible for NACT) unless imaging results indicate otherwise. Additionally, guidelines in BC aim for starting NACT by 7 days after initial medical oncology consultation. This period includes both pre-NACT workup and any wait times due to shortage of chemotherapy chairs, indicating that the latter is likely not a significant barrier to NACT access. While exact numbers are unavailable, local knowledge indicates there are enough medical oncologists to consult with patients seeking NACT in BC in a timely fashion. Therefore, oncologist capacity constraints are unlikely to significantly contribute to NACT delays.

Of the 22% (12/54) of patients whose reasons for delayed NACT were unclear from charts, one possible explanation may be the compounding of wait times both before and after a medical oncology appointment. With a median time of 11 days [IQR 7–16] between first medical oncology consult and beginning NACT, waiting more than just 17 days after diagnosis for a medical oncology consult would be sufficient to delay NACT start past the clinically recommended timeline.

Whether a patient received genetic testing had no correlation with time between diagnosis and beginning NACT. While the diagnosis of a genetic mutation during active cancer treatment may influence surgical decisions, including opting for bilateral mastectomy [24], the waiting period for genetic testing did not seem to influence the timeline for commencing NACT. Although, on average, patients are still waiting more than six months for genetic testing results, they can be obtained within a few weeks when genetic testing can impact surgical decision-making about bilateral mastectomy versus breast-conserving surgery after NACT. Mainstream genetic testing in BC has also reduced wait times from referral to results by over 50% [25]. Similarly, we did not find a correlation between age and time between diagnosis and NACT initiation. As previously mentioned, comorbidities leading to higher NACT risk are more common among older patients, but these do not seem to influence delays to NACT.

No patients explicitly declined or delayed treatment for socioeconomic reasons in our analysis. Previous research has indicated that socioeconomic factors, such as ethnicity, public versus private care, and patient income, may affect access to cancer treatment [26,27,28]. In BC, however, data on ethnicity are not routinely collected in healthcare contexts, limiting our ability to perform related analyses. Additionally, there is no direct financial cost for BC residents to access chemotherapy drugs, which are fully paid for by the government [29]. Employment and disability insurance are available to alleviate indirect financial costs associated with treatment, such as reduced work hours or loss of work, although these costs may still pose a financial burden to patients and their families [30].

Moreover, we did not find a correlation between distance from cancer centre and time to NACT start, as shown in Figure 3. As BC has a large rural population, many patients live far away from cancer centres, where medical oncologists see patients and chemotherapy is often administered. In our study, 21/100 (21%) patients lived more than 50 km in straight-line distance from the nearest cancer centre, with 1 patient living over 1000 km from the nearest cancer centre. These rural patients may face additional barriers to accessing therapy, for example, monetary and financial costs associated with travel, or accommodation costs of staying temporarily near a cancer centre. Nonetheless, no relationship was observed between distance from cancer centre and time from diagnosis to NACT initiation. This may be due to patients receiving chemotherapy through community oncology networks, where chemotherapy can be administered and monitored by a family physician trained in oncology [31].

A 2020 retrospective nationwide analysis in the United States found that the mean time from diagnosis to starting NACT was 35.6 ± 27.5 days, though reasons for delay were not given [32]. In 2023, mean time to medical oncology consultation from referral was 27 days for patients requiring NACT at Vernon Jubilee Hospital in BC [19]. Reasons for omission or delays to NACT other than socioeconomic factors are scarcely documented in the literature. Nonetheless, our findings in this study of a median wait of 40 days [IQR 30–53] from diagnosis to starting NACT and 28 days [IQR 20–49] to a medical oncology consultation are consistent with previous literature and highlight referral to medical oncology as a major barrier to NACT access. Our findings also mark a considerable worsening in wait times for NACT in BC compared to earlier publicly available data. From 2013 to 2018, 82% of patients started NACT within 21 days of diagnosis [18]. Today, just 21% of patients (15/73) start NACT within 28 days of diagnosis.

Based on these findings, the most effective interventions to increase NACT uptake and decrease wait times are those that reduce wait times for an initial medical oncology consultation. Improved triaging could better identify patients potentially eligible for NACT and accelerate their appointment with medical oncology, ensuring that eligible patients are considered for NACT in a timely manner. Integrated multidisciplinary care may also reduce the time from biopsy to NACT initiation [17]. Decreasing wait times to NACT will improve guideline adherence and may ultimately lead to better patient outcomes.

### Study Limitations

The limitations of our study include the small cohort and the limited duration of follow-up. Further work is needed to elucidate reasons for omission or delay of NACT that were not apparent in patient EMRs. As different cancer centres in BC used different EMRs at different timepoints, the number of documents available per patient varied substantially. In particular, patients from the mostly rural Interior and Northern health regions are under-represented in this study due to low uptake of EMRs in these regions and lack of relevant documentation for staging. Despite comprising 20% of the population in BC [33], patients from these health regions represent only 8% of our study cohort. The geographic imbalance in documentation limits our ability to interpret the statistics about NACT access and barriers to accessing NACT among patients residing in rural areas.

## 5. Conclusions

Most eligible patients with high-risk breast cancers in BC receive NACT, but some patients are not referred to medical oncology for consideration of NACT. Furthermore, the majority receive it outside of the clinically recommended window, possibly worsening outcomes. Delays in medical oncology consultations contributed to most cases where delay was observed. Thus, streamlining referrals to medical oncology may help decrease the incidence of both omission and delay of NACT, improving guideline adherence and, potentially, patient outcomes.

### Future Directions

Due to the study cohort being drawn from a brief timeframe, the sample size was limited. Future studies may focus on a larger sample of patients, for example, all eligible patients within a year, and perform subgroup analyses to enable more granular understanding of barriers to NACT access.

## Figures and Tables

**Table 1:** Baseline patient demographics.

Characteristic	*n* = 100
**Health authority (region)**	
Coastal	32
Fraser	31
Interior	3
Island	27
Northern	5
**Distance to cancer centre**	
<10 km	50
10–50 km	29
>50 km	21
**Median age (IQR)**	58.5 [45.3–66]
**Invasive cancer type**	
Ductal	89
Lobular	5
Other	4
Unknown	2
**Clinical stage**	
I	1
II	78
III	20
IV	1
**Biomarker subtype**	
Human epidermal growth factor receptor 2-positive (HER2+)	56
Triple-negative	44
**Grade**	
1	4
2	28
3	55
Unknown	8

**Table 2:** Treatment status of patients.

Characteristic	*n* = 100
**Neoadjuvant chemotherapy (NACT)**	
Recommended by medical oncologist	76
Received	73
Initiated ≤28 days after diagnosis	15
Initiated >28 days after diagnosis	54
Unclear timing	4
Did not receive NACT	27
**Surgery**	99

**Table 3:** Reasons for omission of NACT among eligible patients.

Reason	*n* (%)
**Patient declined NACT offered**	2 (7)
**NACT was not offered**	10 (37)
Comorbidities	5 (19)
NACT of marginal benefit	4 (15)
Patient received “alternative therapy”	1 (4)
**Patient did not see medical oncology before surgery**	8 (30)
Patient not offered medical oncology appointment	7 (26)
Patient declined medical oncology appointment	1 (4)
**Other**	1 (4)
Patient sought treatment out-of-country	1 (4)
Reason unclear on patient chart	6 (22)

**Table 4:** Median time between key steps in the breast cancer treatment pathway.

Timeframe	Median Days [IQR]
**Diagnosis to first consult**	16 [11–26]
**Consult to first treatment**	20 [14–30.5]
**Diagnosis to staging imaging**	20 [13.5–33]
**Diagnosis to first treatment**	40 [30.3–53.8]
**Diagnosis to medical oncology consult**	28 [20–49]
**Diagnosis to chemotherapy**	40 [30–53]
**Diagnosis to surgery consultation**	14 [9–21]
**Diagnosis to surgery**	
Patients receiving NACT	204 [190.5–224.5]
Patients proceeding to surgery first	41.5 [30.5–64.5]
**Medical oncology consultation to NACT**	11 [7–16]

**Table 5:** Main reason for delays in neoadjuvant chemotherapy commencement past 28 days post-diagnosis.

Reason	*n* (%)
**Delay in medical oncologist appointment**	39 (72)
**Patient preference to delay treatment**	1 (2)
**Other patient factors**	1 (2)
**Other provider cause**	1 (2)
**Reason unclear on patient chart**	12 (22)

**Table 6:** Genetic testing status of all patients in the cohort compared to those who received NACT.

Genetic Testing Status	All Patients (*n* = 100) (%)	Received NACT (*n* = 73) (%)
**Genetic testing before surgery**	54 (54)	47 (64)
**Genetic testing after surgery or no genetic testing**	46 (46)	26 (36)

## Data Availability

The data collected in this study contain private health information and are only available on request from the authors after execution of a data-sharing agreement.

## Abbreviations

The following abbreviations are used in the manuscript:

BC	British Columbia
EMR	electronic medical record
HER2	human epidermal growth factor 2
NACT	neoadjuvant chemotherapy
pCR	pathologic complete response
RCB	residual cancer burden
